# Optimal Duration of Ultrasound-Facilitated Catheter-Directed Thrombolysis for Acute Pulmonary Embolism: A Retrospective Cohort Study

**DOI:** 10.3390/biomedicines14091933

**Published:** 2026-08-28

**Authors:** Yu-Kai Lin, Da-Long Chen, Chung-Ho Hsu, Hui-Wen Chang, Keng-Yuan Li, Li-Chuan Hsieh, Chun-Cheng Wang, An-Sheng Lee, Kuan-Cheng Chang

**Affiliations:** 1 Graduate Institute of Biomedical Sciences, China Medical University, Taichung 404327, Taiwan; 2 Division of Cardiovascular Medicine, Department of Medicine, China Medical University Hospital, Taichung 404327, Taiwan; 3 Graduate Institute of Bioinformatics and Medical Engineering, Asia University, Taichung 413305, Taiwan; 4 Department of Pathology, China Medical University Hospital, Taichung 404327, Taiwan; 5 School of Medicine, College of Medicine, China Medical University, Taichung 404327, Taiwan; 6 School of Medicine, Mackay Medical University, New Taipei City 252005, Taiwan; 7 Taichung Municipal Geriatric Rehabilitation General Hospital, Managed by China Medical University, Taichung 406004, Taiwan

**Keywords:** acute pulmonary embolism, tissue-type plasminogen activator, ultrasound-facilitated catheter-directed thrombolysis, EkoSonic Endovascular System

## Abstract

**Background/Objectives:** The mortality rate for acute high-risk and intermediate–high-risk pulmonary embolism (PE) was high despite treatment with heparin anticoagulation alone. Although adjunctive systemic thrombolysis can significantly reduce mortality, it still increases the incidence of major bleeding. Ultrasound-facilitated catheter-directed thrombolysis (USCDT) has been shown to reduce the incidence of major bleeding; however, the optimal treatment duration remains uncertain. **Methods:** This retrospective cohort study included 155 hospitalized patients with acute PE who underwent USCDT between January 2017 and June 2025 in the intensive care unit at China Medical University Hospital, Taichung, Taiwan. Patients were categorized into four groups based on average usage time: <20, 20–29, 30–39, and ≥40 h. The primary endpoints were 30-day all-cause mortality rate and major bleeding. Covariates associated with 30-day all-cause mortality were estimated and adjusted using logistic regression modeling. **Results:** Across all treatment groups, USCDT significantly reduced systolic pulmonary artery (PA) pressure (26.9%), mean PA pressure (21.0%), fibrinogen levels (25.2%), and right ventricle-to-left ventricle diameter ratio (21.5%) (all *p* < 0.001). Compared with the group with a USCDT ≥ 40 h, the group with a USCDT < 20 h had a lower odds ratio for 30-day all-cause mortality (OR: 0.36, 95% CI: 0.07–1.81). A similar benefit was observed for a USCDT 20–29 h. **Conclusions:** USCDT < 20 or 20–29 h (mean 15–20 mg alteplase) may provide an optimal balance between efficacy and safety by reducing 30-day all-cause mortality and major bleeding in patients with acute PE.

## 1. Introduction

Acute pulmonary embolism (PE) is characterized by the acute dislodgement of a thrombus formed in a deep vein into the pulmonary artery (PA), resulting in acute hypoxemia and right ventricular strain. Although the incidence of pulmonary embolism (PE) is 14 cases per 100,000 people and the average mortality rate is 7%, the mortality rate for high-risk acute PA reaches 30–50%, while that for intermediate–high-risk PE is 10–15% [1,2]. Therefore, the European Society of Cardiology defines high-risk PE as persistent hypotension lasting more than 15 min or any signs of shock, such as altered mental status, oliguria, or elevated lactate levels. Intermediate–high-risk PE is defined as right ventricular dysfunction, characterized by a right ventricle-to-left ventricle (RV/LV) diameter ratio greater than 1 on imaging and elevated cardiac troponin-I levels [3].

Systemic thrombolytic therapy, supplemented with baseline heparin anticoagulation, has been the primary reperfusion treatment for high-risk PE and a rescue reperfusion strategy for intermediate–high-risk PE for more than a decade [4]. This approach has reduced the mortality rate to 15–20% in high-risk acute PE and 5–7% in intermediate–high-risk PE. However, systemic thrombolysis is associated with an increased risk of major bleeding (10–20%), particularly intracranial hemorrhage, which occurs in 2–5% of cases [5]. The 2026 clinical practice guidelines published by the Joint Committee of the American College of Cardiology and the American Heart Association recommend early systemic thrombolysis for primary reperfusion in any type of shock and for rescue reperfusion in any episode of transient hypotension or other signs of hemodynamic instability [6].

Catheter-directed thrombolysis (CDT), which delivers thrombolytic agents directly into the PA through side-hole catheters, provides efficacy comparable to that of systemic thrombolysis while reducing the incidence of major bleeding to 7–15% and intracranial hemorrhage to 1–3%, resulting in an overall 30% reduction in bleeding complications [7,8]. Consequently, CDT has become a viable alternative to systemic thrombolysis, particularly for patients with PE who have not experienced cardiac arrest [9]. Ultrasound-facilitated catheter-directed thrombolysis (USCDT), performed using the EkoSonic Endovascular System, further enhances thrombolytic delivery by facilitating more precise administration of tissue-type plasminogen activator (alteplase) and has gradually replaced conventional CDT in recent years [10,11,12]. USCDT has demonstrated efficacy in patients with both high-risk and intermediate–high-risk PE [13,14].

Although low-dose alteplase administered with USCDT has been shown to be effective in the treatment of PE [15], the optimal alteplase dose in combination with the EkoSonic Endovascular System treatment duration remains unclear. Therefore, this study aimed to determine the optimal USCDT treatment duration.

## 2. Materials and Methods

### 2.1. Study Design and Population

Data were retrieved from the electronic medical records of China Medical University Hospital. The requirement for written informed consent was waived due to the retrospective study design. The study was approved by the Institutional Review Board of China Medical University Hospital (CMUH114-REC3-201) and was conducted in accordance with its ethical guidelines.

**Figure 1:**
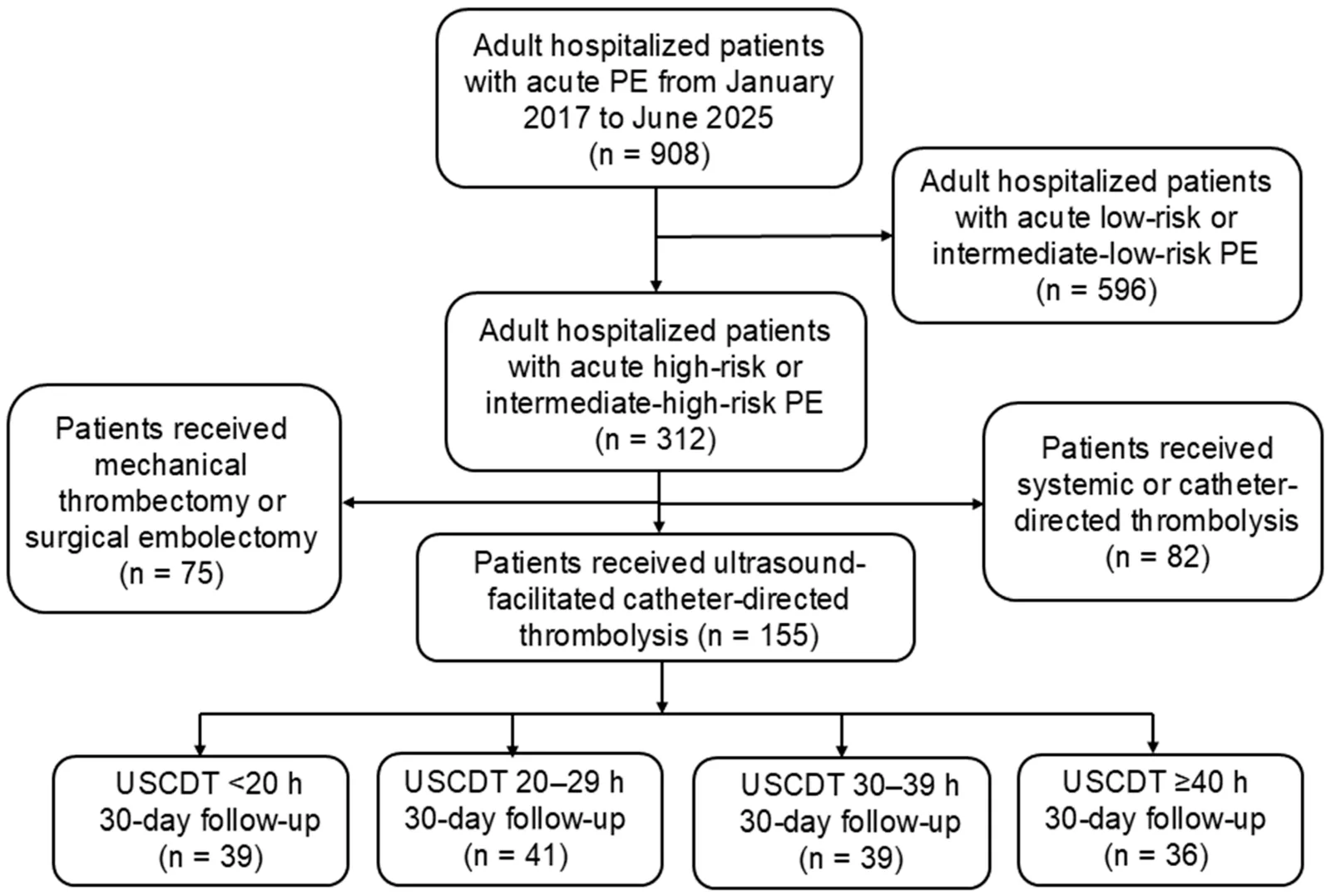
STROBE flowchart for high-risk or intermediate–high-risk PE patients who underwent USCDT. A total of 908 adult patients with PE, hospitalized between January 2017 and June 2025, were enrolled for further evaluation. Subsequently, 596 patients with low-risk or intermediate–low-risk PE were excluded, and 312 patients with high-risk or intermediate–high-risk PE were enrolled. Patients who received mechanical thrombectomy or surgical embolectomy were excluded, as were those who received systemic thrombolysis or conventional CDT. Finally, the 155 eligible patients who underwent USCDT were analyzed. We divided them into 4 groups according to the duration of USCDT. The outcomes assessed included mortality and major bleeding after 30-day follow-up. PE, pulmonary embolism; STROBE, Strengthening the Reporting of Observational Studies; USCDT, ultrasound-facilitated, catheter-directed thrombolysis.

This observational, retrospective cohort study included 155 consecutive adult patients with acute high-risk or intermediate–high-risk PE who underwent USCDT and were admitted to the intensive care unit of China Medical University Hospital, Taichung, Taiwan, between January 2017 and June 2025. Patients with acute low-risk or intermediate–low-risk PE were excluded. Patients who underwent mechanical thrombectomy, surgical embolectomy, systemic thrombolysis, or conventional CDT were also excluded (STROBE flowchart; Figure 1). The remaining 155 patients were categorized into four groups according to USCDT duration: <20, 20–29, 30–39, and ≥40 h. All patients were followed for 30 days to assess all-cause mortality and major bleeding.

### 2.2. Definitions of Acute Pulmonary Embolism, Primary Thrombolysis, and Rescue Thrombolysis

Acute PE was defined as symptom onset (e.g., dyspnea or palpitations) occurring within 14 days before presentation and a thrombus observed in the PA confirmed by computed tomography pulmonary angiography (CTPA). Primary thrombolysis was performed within 1–3 h of diagnosis in patients with acute high-risk PE, whereas rescue thrombolysis was performed within 24–48 h after initiation of heparin anticoagulation in patients with acute intermediate–high-risk PE.

### 2.3. Endovascular Ultrasound and Thrombolysis with Tissue-Type Plasminogen Activator

The EkoSonic Endovascular System (Boston Scientific, Marlborough, MA, USA) is a commercially available endovascular ultrasound used for CDT. The system utilizes acoustic microstreaming generated by 200 Hz ultrasound to loosen and separate tightly entangled fibrin mesh fibers, a process known as “unwinding fibrin mesh.” This process exposes additional fibrin-binding sites and enhances thrombolytic drug penetration.

Alteplase was used to convert plasminogen to plasmin. Plasmin then cleaves the cross-linked fibrin mesh into smaller, soluble fragments, such as D-dimers.

### 2.4. Anticoagulation with Heparin

Heparin exerts its anticoagulant effect through antithrombin III. After binding to heparin, antithrombin III further binds to coagulation factors XII, XI, IX, and X, as well as thrombin. The heparin/antithrombin III complex inactivates coagulation factor X and thrombin, thereby inhibiting thrombin formation and the conversion of fibrinogen to fibrin. After 5–10 days of continuous heparin infusion, patients were transitioned to novel oral anticoagulants (NOACs), which directly inhibit factor X or thrombin, for 3–6 months. Patients who could not tolerate NOACs were transitioned to vitamin K antagonists.

### 2.5. Ultrasound-Facilitated Catheter-Directed Thrombolysis and Anticoagulation Protocol at China Medical University Hospital

USCDT treatment protocol: alteplase of 2–4 mg was injected into the PA, followed by continuous infusion at a rate of 1 mg/h for 12–48 h through a side-hole catheter placed in the PA. Fibrinogen levels were measured every 6 h; if the fibrinogen level fell below 200 mg/dL, the infusion of alteplase was suspended. An ultrasound device was connected to the EkoSonic Endovascular System to enhance treatment efficacy.

Anticoagulation treatment regimen: A heparin loading dose of 60 U/kg was administered, followed by continuous infusion to maintain the activated partial thromboplastin time between 50 and 70 s for 5–10 days. While used in combination with alteplase, the target activated partial thromboplastin time was maintained between 45 and 60 s.

### 2.6. Primary and Secondary Outcomes

The primary outcomes were 30-day all-cause mortality and major bleeding. Major bleeding was defined according to the Bleeding Academic Research Consortium (BARC) Scale as type 3B (hemoglobin decrease >5 g/dL from baseline or bleeding requiring surgical intervention), type 3C (critical condition, such as intracranial or intraocular hemorrhage), type 4 (bleeding related to coronary artery bypass graft procedures), or type 5 (fatal bleeding confirmed by imaging or autopsy).

The secondary outcomes included 48 h changes in PA systolic pressure, PA mean pressure, fibrinogen concentration, and the RV/LV diameter ratio after the procedure.

### 2.7. Statistical Analysis

In order to collect data, we need to calculate the sample size in advance. The sample size was calculated to detect a clinically significant 75% reduction in the primary outcome between the high-risk PE group (20%) and intermediate–high-risk PE group (5%) to ensure that this included cohort had sufficient statistical power. A total of 150 patients (75 per group) was required to achieve 80% power with a two-sided significance level of 0.05, assuming that 20% of cases would be non-evaluable.

For post hoc analysis, continuous variables are presented as the mean ± standard deviation and were compared using one-way analysis of variance. Categorical variables are expressed as frequencies and percentages and were compared using Pearson’s chi-square test. Student’s *t*-test was used for pairwise comparisons, where appropriate. A two-sided *p*-value of <0.05 was considered statistically significant. The odds ratio of 30-day all-cause mortality, 95% confidence intervals, and related significant values were obtained from the multivariable logistic regression analysis. All statistical analyses were performed using SPSS Statistics, version 30.0 (IBM Corp., Armonk, NY, USA).

## 3. Results

### 3.1. Baseline Clinical Characteristics

Table 1 summarizes the baseline clinical characteristics of the study participants, categorized according to USCDT treatment duration. Baseline clinical characteristics were similar across all four duration groups (USCDT < 20, 20–29, 30–39, and ≥40 h), with no statistically significant differences. The mean age at diagnosis was 59 ± 19 years, and most patients were female (67.7%). The most prevalent comorbidities (>10%) were deep vein thrombosis (55.5%), hypertension (32.9%), malignancy (27.7%), diabetes mellitus (20.0%), immobility (19.4%), surgery (13.5%), and autoimmune disease (12.3%).

Regarding PE severity, 44.5% of patients had high-risk PE and 55.5% had intermediate–high-risk PE. The Pulmonary Embolism Severity Index (PESI) was 115.6 ± 37.0, and the Mastora obstruction index was 44.2 ± 8.4%. Regarding adjunctive procedures, 21.3% of patients received extracorporeal membrane oxygenation (ECMO) because of shock. After thrombolysis or ECMO removal, 66.5% of patients received an inferior vena cava filter for 1–3 months.

### 3.2. Follow-Up of Laboratory Tests on the Day of Admission

Table 2 shows the laboratory findings on the day of admission. Laboratory findings were similar across all duration groups (USCDT < 20, 20–29, 30–39, and ≥40 h), with no statistically significant differences. For blood counts, the mean white blood cell count was 11.9 ± 6.3 K/μL, hemoglobin was 12.4 ± 2.1 g/dL, and platelet count was 202.5 ± 83.8 K/μL. For biochemical indices, the mean troponin-I level was 0.35 ± 1.13 ng/mL, estimated glomerular filtration was 70.6 ± 33.2 mL/min/1.73 m^2^, and lactate level was 2.2 ± 1.0 mmol/L. Elevated troponin-I (>0.04 ng/mL) and lactate (>2.0 mmol/L) levels were observed in 67.7% and 44.5% of patients, respectively.

### 3.3. Secondary-Outcome Analyses of Treatment Effect on Pulmonary Artery Systolic and Mean Pressure, Fibrinogen Concentration, and the Right Ventricle-to-Left Ventricle Diameter Ratio

**Figure 2:**
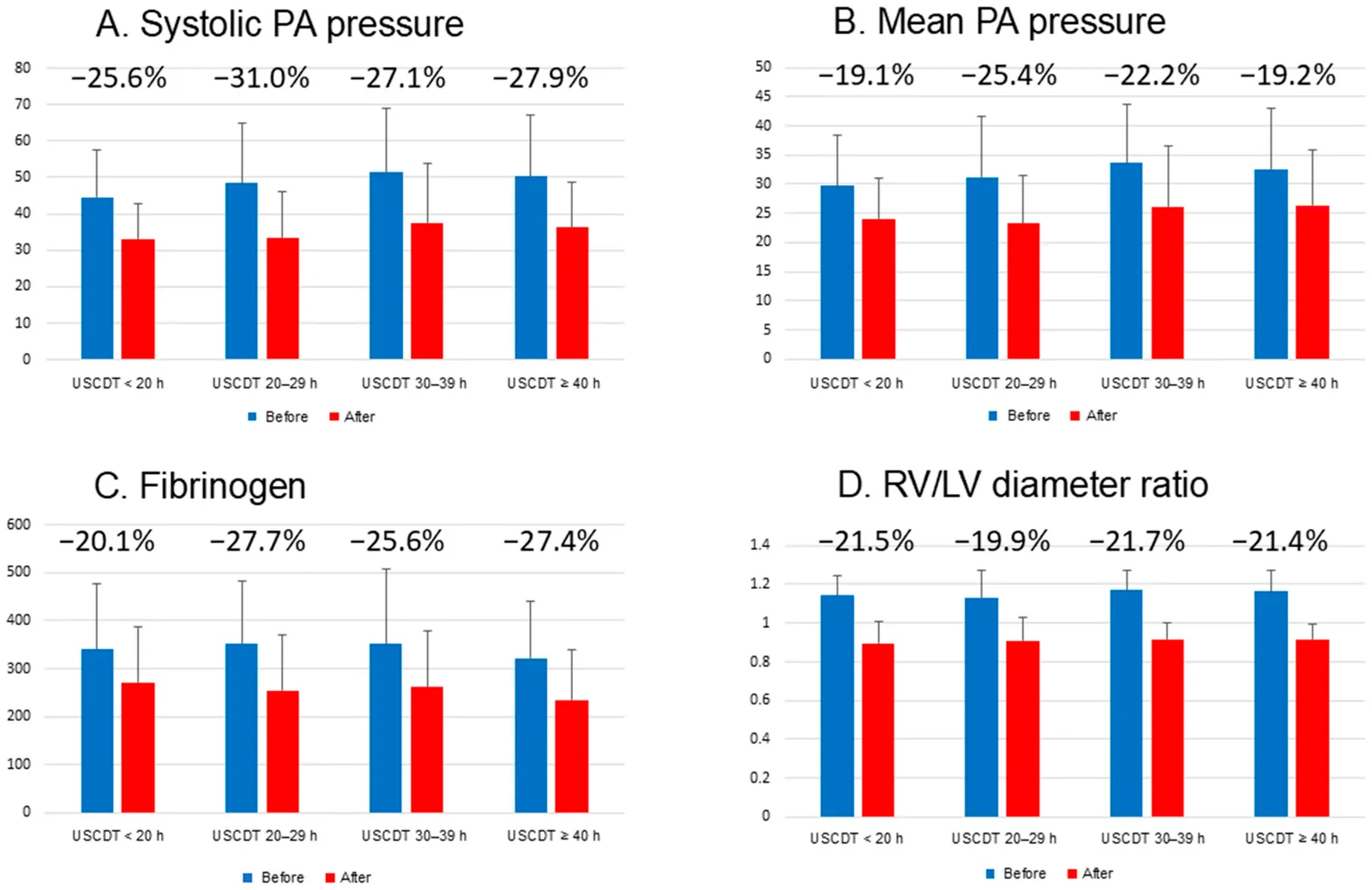
After 48 h of follow-up with a mean duration of USCDT of 34.2 h and a mean dose of alteplase of 26.8 mg: (**A**) systolic PA pressure decreased from 48.7 to 35.1 mmHg (a change of 26.9%, *p* < 0.001); (**B**) mean PA pressure decreased from 31.8 to 24.9 mmHg (a change of 21.0%, *p* < 0.001); (**C**) fibrinogen decreased from 341.9 to 255.8 mg/dL (a change of 25.2%, *p* < 0.001); and (**D**) RV/LV diameter ratio decreased from 1.15 to 0.91 (change of 21.5%, *p* < 0.001). PA, pulmonary artery; RV/LV, right ventricle to left ventricle. Unit of PA pressure: mmHg; unit of fibrinogen concentration: mg/dL.

Table 3 and Figure 2 present the 48 h treatment effects of different USCDT treatment durations. The group of USCDT < 20 h had a mean treatment duration of 12 h and a mean alteplase dose of 15 mg. The group of USCDT 20–29 h had a mean treatment duration of 24 h and a mean alteplase dose of 20 mg. The group of USCDT 30–39 h had a mean treatment duration of 36 h and a mean alteplase dose of 31 mg. The group of USCDT ≥ 40 h had a mean treatment duration of 67 h and a mean alteplase dose of 43 mg. All four groups demonstrated favorable secondary outcomes, with no statistically significant differences among the groups. After 48 h of follow-up, with a mean USCDT treatment duration of 34 h and a mean alteplase dose of 27 mg, systolic PA pressure decreased from 48.7 to 35.1 mmHg (26.9%, *p* < 0.001), mean PA pressure decreased from 31.8 to 24.9 mmHg (21.0%, *p* < 0.001), fibrinogen concentration decreased from 341.9 to 255.8 mg/dL (25.2%, *p* < 0.001), and RV/LV diameter ratio decreased from 1.15 to 0.91 (21.5%, *p* < 0.001).

### 3.4. Primary-Outcome Analyses of Treatment Effect on Major Bleeding and 30-Day All-Cause Mortality

Table 4 summarizes the primary clinical outcomes, including major bleeding and 30-day all-cause mortality. Compared with the group with a USCDT of ≥40 h, the group with a USCDT of <20 h had a relatively lower rate of major bleeding (10.3% vs. 16.7%, trend *p*-value = 0.36). This major bleeding was primarily of the BARC 3B type (7.7% vs. 13.9%, trend *p*-value = 0.40), with the exception of BARC 3C bleeding (2.6% vs. 2.8%, trend *p*-value = 0.75). Compared with the regimen of USCDT ≥ 40 h, the regimen of USCDT < 20 h showed a trend toward lower 30-day all-cause mortality (5.1% vs. 13.9%, trend *p*-value = 0.14).

**Figure 3:**
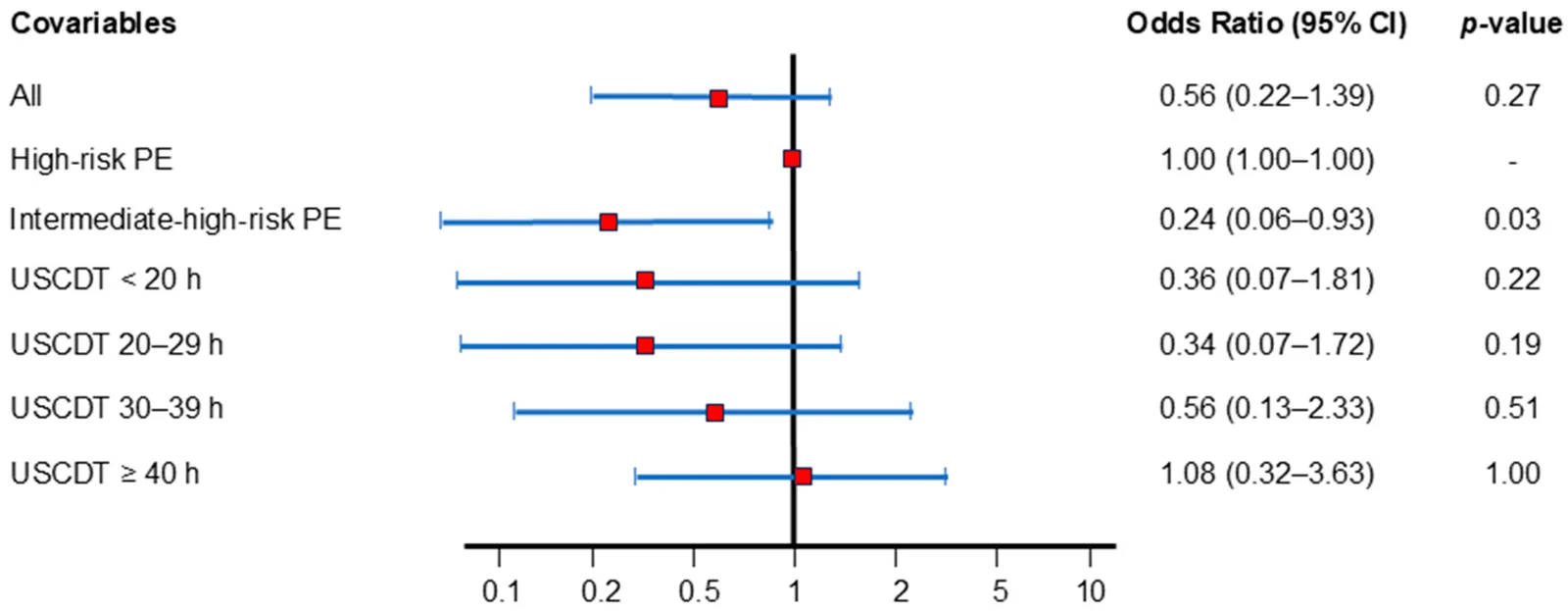
Thirty-day mortality rate of different PE severity or USCDT duration using logistic regression model. High-risk PE after thrombolysis was used as a reference point for 30-day mortality. Covariables include all PE after thrombolysis (odds ratio: 0.56, 95% CI: 0.22–1.39), intermediate–high-risk PE after thrombolysis (odds ratio: 0.24, 95% CI: 0.06–0.93), USCDT < 20 h (odds ratio: 0.36, 95% CI: 0.07–1.81), USCDT 20–29 h (odds ratio: 0.34, 95% CI: 0.07–1.72), USCDT 30–39 h (odds ratio: 0.56, 95% CI: 0.13–2.33), and USCDT ≥ 40 h (odds ratio: 1.08, 95% CI: 0.32–3.63). PE, pulmonary embolism; USCDT, ultrasound-facilitated catheter-directed thrombolysis.

Figure 3 illustrates several covariates analyzed as protective factors against 30-day all-cause mortality after thrombolysis. High-risk PE after thrombolysis was used as the reference point for 30-day all-cause mortality. Covariates included all PE after thrombolysis (odds ratio: 0.56, 95% CI: 0.22–1.39), intermediate–high-risk PE after thrombolysis (odds ratio: 0.24, 95% CI: 0.06–0.93), and four grouped protective factors as follows: USCDT < 20 h (odds ratio: 0.36, 95% CI: 0.07–1.81); USCDT 20–29 h (odds ratio: 0.34, 95% CI: 0.07–1.72); USCDT 30–39 h (odds ratio: 0.56, 95% CI: 0.13–2.33); and USCDT ≥ 40 h (odds ratio: 1.08, 95% CI: 0.32–3.63).

## 4. Discussion

### 4.1. Principal Findings

In this study, we evaluated potential protective factors associated with improved 30-day all-cause mortality after USCDT in patients with acute high-risk or intermediate–high-risk PE. In this retrospective study, the optimal USCDT duration was <20 or 20–29 h, corresponding to a mean alteplase dose of 15–20 mg. Prolonged USCDT ≥ 40 h (mean 43 mg of alteplase) did not provide additional clinical benefits and was associated with a potential risk of major bleeding and an upward numerical trend in 30-day all-cause mortality.

### 4.2. Mechanism of Thrombolysis with Tissue-Type Plasminogen Activator

In acute PE, thrombin catalyzes the conversion of fibrinogen into fibrin monomers. Large amounts of fibrin monomers bind to factor XIII to form X-chain fibrin, known as a fibrin mesh. This fibrin mesh traps red blood cells, forming a red thrombus. Heparin anticoagulation primarily reduces thrombin formation and further promotes endogenous fibrinolysis after 48 h. However, in critical situations, such as high-risk or intermediate–high-risk PE, alteplase should be administered to trigger plasmin formation within 3 h for high-risk PE or within 48 h for intermediate–high-risk PE. Plasmin rapidly breaks down the fibrin mesh, producing large amounts of D-dimers. Importantly, a decrease in fibrinogen concentration has consistently been an indicator of the effectiveness of thrombolytic therapy because this treatment simultaneously reduces both fibrinogen and fibrin concentrations [15]. Fibrinogen is a marker of both thrombosis and bleeding; when fibrinogen levels fall below 200 mg/dL, platelet aggregation becomes impaired. Consequently, damage to blood vessels may result in loss of the coagulation response, leading to major bleeding.

### 4.3. Right-Heart Function Improved by Ultrasound-Facilitated Catheter-Directed Thrombolysis

USCDT is typically performed using the EkoSonic Endovascular System. Many clinical trials have demonstrated that continuous alteplase infusion for either standard or extended durations reduces systolic and mean PA pressures and the RV/LV diameter ratio, thereby improving right-heart function [16,17,18]. In our study, after 48 h of USCDT, PA systolic pressure decreased from 48.7 to 35.1 mmHg (26.9%, *p* < 0.001), PA mean pressure decreased from 31.8 to 24.9 mmHg (21.0%, *p* < 0.001), and the RV/LV diameter ratio decreased from 1.15 to 0.91 (21.5%, *p* < 0.001). These findings are highly consistent with those of previous USCDT studies [17,18]. If the thrombus in the PA rapidly dissolves, the hypoxic conditions will improve. As pulmonary vascular resistance decreases rapidly, PA systolic and mean pressures naturally drop. A reduction in PA pressure not only improves RV-PA coupling but also reduces RV afterload. As RV contractility improves, the RV/LV diameter ratio decreases accordingly, facilitating recovery of right-heart function.

### 4.4. Duration of Ultrasound-Facilitated Catheter-Directed Thrombolysis Researched

Ten years ago, a series of clinical trials evaluated the treatment duration for USCDT. The SEATTLE-II study investigated USCDT with different treatment durations: 12 h for bilateral PA treatment and 24 h for unilateral PA treatment, with a total alteplase dose of 24 mg [19]. The ULTIMA study, on the other hand, examined USCDT with different doses of alteplase: a dose of 10 mg for unilateral treatment and 20 mg for bilateral treatment, both administered over a 15 h treatment period [20]. All treatment regimens demonstrated similar efficacy and safety profiles. In recent years, researchers have attempted to use shorter treatment durations and lower alteplase doses (e.g., 2–6 h, with doses of 4–6 mg for unilateral treatment and 8–12 mg for bilateral treatment). Although these shorter treatment regimens are primarily intended for intermediate–high-risk PE, they have also demonstrated comparable efficacy and safety [21,22].

### 4.5. Prolonged Duration of Ultrasound-Facilitated Catheter-Directed Thrombolysis Increases the Potential Risks of Major Bleeding

For patients with high-risk or intermediate–high-risk PE, attempts have been made to extend treatment duration and increase the t-PA dose. One study reported that despite doubling the alteplase dose, no significant increase in major bleeding occurred [23]. However, in this study, USCDT < 20 h had a relatively lower incidence of major bleeding (10.3% vs. 16.7%, trend *p*-value = 0.36) compared with that performed using USCDT ≥ 40 h, comprising mainly BARC type 3B bleeding (7.7% vs. 13.9%, trend *p*-value = 0.40), with the exception of BARC type 3C bleeding (2.6% vs. 2.8%, trend *p*-value = 0.75). These findings are consistent with previous clinical observations. The incidence of intracranial hemorrhage (BARC type 3C) remained low (1–3%) and appears to be more related to underlying conditions (e.g., history of stroke or uncontrolled blood pressure) rather than alteplase dose [24]. Regarding underlying factors, while bleeding complications showed no statistically significant correction with mortality, their potential clinical contribution to adverse outcomes cannot be entirely ruled out.

### 4.6. Pathophysiological Mechanisms of Prolonged Duration of Ultrasound-Facilitated Catheter-Directed Thrombolysis

Furthermore, we hypothesize that rapid restoration of blood flow post-thrombolysis may induce ischemic reperfusion injury, triggering systemic inflammation and organ dysfunction, which could serve as another critical pathophysiological driver of mortality. Although myocardial infarction or cardiac arrest are the primary causes of mitochondrial calcium overload, acute PE primarily affects the lungs, which have a dual arterial blood supply including the PA and bronchial arteries that makes them more susceptible to ferroptosis during reperfusion [25,26]. Alveolar epithelial and endothelial cells are directly exposed to high oxygen concentrations; consequently, the local production of reactive oxygen species (ROS) during reperfusion is far more intense than that in other organs. Furthermore, the lungs contain large amounts of pulmonary surfactant and complex cell membrane structures rich in highly sensitive polyunsaturated fatty acids. Under intense ROS attack, these lipids are highly susceptible to chain oxidation reactions. During ischemia–reperfusion injury, pulmonary microcirculation is often accompanied by increased microvascular permeability, red blood cell extravasation, and hemolysis, leading to the release of large amounts of free iron. The combination of high iron and ROS levels rapidly triggers the Fenton reaction, generating highly destructive hydroxyl radicals that directly accelerate the accumulation of lipid hydroperoxides [26,27]. This further illustrates that prolonging the thrombolysis time not only fails to provide additional therapeutic benefits, but also significantly increases reperfusion injury, leading to thrombogenesis [28,29], endothelial dysfunction, and multiple organ failure [30,31].

### 4.7. Prolonged Duration of Ultrasound-Facilitated Catheter-Directed Thrombolysis Increased the Numerical Trend of Mortality

Both the potential risk of major bleeding and reperfusion injury can lead to multiple organ failure, thereby contributing to mortality. We analyzed several covariates—based on the severity or duration of USCDT—as protective factors for 30-day all-cause mortality following thrombolytic therapy. Using high-risk PE as the reference group, intermediate–high-risk PE after thrombolysis was associated with a lower risk of 30-day all-cause mortality (odds ratio: 0.24, 95% CI: 0.06–0.93, *p* = 0.03), supporting the reliability of this analysis. We further evaluated protective factors based on USCDT duration. Compared with thrombolytic therapy using USCDT ≥ 40 h (odds ratio: 1.08, 95% CI: 0.32–3.63, *p* = 1.00), thrombolytic therapy using USCDT < 20 h was associated with a relatively lower odds ratio for 30-day all-cause mortality (odds ratio: 0.36, 95% CI: 0.07–1.81, *p* = 0.22). Although no statistically significant differences were observed, the numerical trend of mortality rates increased with longer USCDT durations, strongly suggesting that shorter USCDT durations may be preferable to longer ones.

### 4.8. Limitations

This study has several limitations. First, the RV/LV diameter ratio was measured using CTPA before USCDT but by standard echocardiography 48 h after USCDT. The use of different imaging modalities may have introduced measurement variability. Second, this was a single-center study, which may limit the generalizability of the findings. Third, the role of ischemia–reperfusion injury remains hypothetical and requires validation using animal models. Finally, prospective randomized controlled trials are needed for the two groups with USCDT < 20 and 20–29 h to validate these findings and further determine the optimal duration of USCDT treatment.

## 5. Conclusions

In this study, USCDT < 20 h (mean alteplase dose of 15 mg) was associated with a relatively lower odds ratio for 30-day all-cause mortality (odds ratio: 0.36; 95% CI, 0.07–1.81, *p* = 0.22) than prolonged-duration USCDT ≥ 40 h (odds ratio: 1.08; 95% CI, 0.32–3.63, *p* = 1.00), although the difference was not statistically significant. USCDT 20–29 h (mean alteplase dose of 20 mg) also showed results very similar to those observed with USCDT < 20 h. These findings suggest that USCDT < 20 or 20–29 h may provide an optimal balance between efficacy and safety in patients with acute high-risk or intermediate–high-risk PE by reducing 30-day all-cause mortality and major bleeding.

## Figures and Tables

**Table 1:** Baseline clinical characteristics of study subjects depending on duration of USCDT.

	USCDT < 20 h (n = 39)	USCDT 20–29 h (n = 41)	USCDT 30–39 h (n = 39)	USCDT ≥ 40 h (n = 36)	*p*-Value for Trend
**Age (y), mean ± SD**	63 ± 19	58 ± 20	57 ± 17	58 ± 19	0.27
**Female sex, n (%)**	31 (79.5)	23 (56.1)	25 (64.1)	26 (72.2)	0.67
**Body mass index (kg/m^2^), mean ± SD**	25.3 ± 3.7	26.1 ± 5.7	26.1 ± 4.8	26.8 ± 7.4	0.26
**Vital signs**					
**Heart rate, mean ± SD**	95.5 ± 22.9	96.3 ± 21.1	92.7 ± 18.3	104.3 ± 18.0	0.12
**Systolic blood pressure, mean ± SD**	121.7 ± 18.9	122.5 ± 16.7	122.1 ± 14.3	113.7 ± 16.0	0.05
**Diastolic blood pressure, mean ± SD**	76.0 ± 13.7	75.0 ± 12.4	76.5 ± 13.1	72.8 ± 11.7	0.40
**Oxygen saturation, %**	89.6	87.7	88.2	87.8	0.12
**Chronic disease**					
**Pregnancy, n (%)**	1 (2.6)	4 (9.8)	1 (2.6)	2 (5.6)	0.93
**Smoking, n (%)**	2 (5.1)	4 (9.8)	3 (7.7)	3 (8.3)	0.70
**Surgery, n (%)**	5 (12.8)	8 (19.5)	5 (12.8)	3 (8.3)	0.43
**Immobility, n (%)**	9 (23.1)	4 (9.8)	8 (20.5)	9 (25.0)	0.57
**Atrial fibrillation, n (%)**	3 (7.7)	2 (4.9)	2 (5.1)	1 (2.8)	0.37
**Diabetes mellitus, n (%)**	8 (20.5)	7 (17.1)	9 (23.1)	7 (19.4)	0.91
**Hypertension, n (%)**	13 (33.3)	14 (34.2)	13 (33.3)	11 (30.6)	0.79
**Coronary artery disease, n (%)**	1 (2.6)	1 (2.4)	3 (7.7)	2 (5.6)	0.34
**Malignancy, n (%)**	11 (28.2)	10 (24.4)	10 (25.6)	12 (33.3)	0.62
**Heart failure, n (%)**	1 (2.6)	2 (4.9)	4 (10.3)	1 (2.8)	0.67
**Stroke, n (%)**	2 (5.1)	3 (7.3)	5 (12.8)	4 (11.1)	0.26
**Autoimmune disease, n (%)**	2 (5.1)	5 (12.2)	5 (12.8)	7 (19.4)	0.07
**Deep vein thrombosis, n (%)**	18 (46.2)	23 (56.1)	20 (51.3)	25 (69.4)	0.08
**Severity assessment**					
**High risk, n (%)**	14 (35.9)	16 (39.0)	19 (48.7)	20 (55.6)	0.06
**PESI, mean ± SD**	110.9 ± 42.1	114.0 ± 32.8	118.4 ± 39.3	119.3 ± 33.5	0.28
**Mastora obstruction index, %**	42.9 ± 8.4	44.8 ± 8.6	44.6 ± 9.0	44.7 ± 7.5	0.39
**Additional procedure**					
**ECMO, n (%)**	8 (20.5)	5 (12.2)	10 (25.6)	10 (27.8)	0.24
**Inferior vena cava filter, n (%)**	26 (66.7)	32 (78.0)	24 (61.5)	21 (58.3)	0.23

ECMO, extracorporeal membrane oxygenation; PESI, Pulmonary Embolism Severity Index; SD, standard deviation; USCDT, ultrasound-facilitated catheter-directed thrombolysis.

**Table 2:** Baseline blood counts and biochemical indices of study subjects depending on duration of USCDT.

	USCDT < 20 h (n = 39)	USCDT 20–29 h (n = 41)	USCDT 30–39 h (n = 39)	USCDT ≥ 40 h (n = 36)	*p*-Value for Trend
**Blood counts**					
White blood cells (K/μL), mean ± SD	12.6 ± 8.0	11.3 ± 4.5	12.0 ± 6.3	11.8 ± 5.9	0.69
Hemoglobin (g/dL), mean ± SD	12.0 ± 2.2	12.9 ± 1.9	12.8 ± 2.2	12.0 ± 1.9	0.90
Platelets (K/μL), mean ± SD	217.8 ± 86.6	188.9 ± 59.9	192.2 ± 89.5	212.6 ± 96.3	0.84
**Biochemical indices**					
Troponin-I (ng/mL), mean ± SD	0.24 ± 0.42	0.44 ± 1.48	0.28 ± 0.73	0.45 ± 1.53	0.58
Troponin-I > 0.04 ng/mL, n (%)	31 (79.5)	24 (58.5)	24 (61.5)	26 (72.2)	0.56
eGFR (mL/min), mean ± SD	70.8 ± 33.6	71.9 ± 35.7	66.9 ± 31.5	72.9 ± 32.6	0.95
Lactate (mmol/L), mean ± SD	2.0 ± 1.0	2.1 ± 1.0	2.4 ± 1.0	2.3 ± 0.9	0.19
Lactate > 2.0 mmol/L, n (%)	14 (35.9)	16 (39.0)	19 (48.7)	20 (55.6)	0.06

eGFR, estimated glomerular filtration rate; USCDT, ultrasound-facilitated catheter-directed thrombolysis.

**Table 3:** Different duration of USCDT effect in study subjects with acute high-risk and intermediate–high-risk PE.

	USCDT < 20 h (n = 39)	USCDT 20–29 h (n = 41)	USCDT 30–39 h (n = 39)	USCDT ≥ 40 h (n = 36)	*p*-Value for Trend
Duration (h), mean ± SD	12.2 ± 5.9	24.4 ± 3.0	36.4 ± 2.8	66.9 ± 23.1	<0.001
Alteplase dose (mg), mean ± SD	14.6 ± 9.9	20.3 ± 11.3	31.2 ± 14.4	42.6 ± 30.1	<0.001
**Systolic PA pressure**					
Systolic PA pressure (before), mean ± SD	44.5 ± 13.1	48.5 ± 16.6	51.6 ± 17.3	50.2 ± 16.9	0.09
Systolic PA pressure (after), mean ± SD	33.1 ± 9.5	33.5 ± 12.5	37.6 ± 16.2	36.2 ± 12.4	0.16
Change from baseline at 48 h (%)	25.6	31.0	27.1	27.9	
*p*-value of change rate	<0.001	<0.001	<0.001	<0.001	
**Mean PA pressure**					
Mean PA pressure (before), mean ± SD	29.7 ± 8.6	31.2 ± 10.3	33.6 ± 10.2	32.6 ± 10.3	0.13
Mean PA pressure (after), mean ± SD	24.1 ± 6.9	23.3 ± 8.2	26.1 ± 10.4	26.3 ± 9.4	0.13
Change from baseline at 48 h (%)	19.1	25.4	22.2	19.2	
*p*-value of change rate	0.002	<0.001	0.002	0.009	
**Fibrinogen concentration**					
Fibrinogen (before), mean ± SD	339.8 ± 138.2	352.6 ± 130.9	350.8 ± 157.3	322.4 ± 118.9	0.59
Fibrinogen (after), mean ± SD	271.7 ± 114.3	254.9 ± 114.4	261.0 ± 117.8	234.2 ± 103.9	0.20
Change from baseline at 48 h (%)	20.1	27.7	25.6	27.4	
*p*-value of change rate	0.02	0.001	0.006	0.001	
**RV/LV diameter ratio**					
RV/LV diameter ratio (before), mean ± SD	1.14 ± 0.11	1.13 ± 0.14	1.17 ± 0.10	1.16 ± 0.11	0.17
RV/LV diameter ratio (after), mean ± SD	0.89 ± 0.11	0.91 ± 0.12	0.92 ± 0.09	0.92 ± 0.08	0.47
Change from baseline at 48 h (%)	21.5	19.9	21.7	21.4	
*p*-value of change rate	<0.001	<0.001	<0.001	<0.001	

RV/LV, right ventricle-to-left ventricle; SD, standard deviation; USCDT, ultrasound-facilitated catheter-directed thrombolysis. Unit of PA pressure: mmHg; unit of fibrinogen concentration: mg/dL.

**Table 4:** Clinical outcomes of study subjects depending on different durations of USCDT.

	USCDT < 20 h (n = 39)	USCDT 20–29 h (n = 41)	USCDT 30–39 h (n = 39)	USCDT ≥ 40 h (n = 36)	*p*-Value for Trend
**Major complications**					
Major bleeding, n (%)	4 (10.3)	4 (9.8)	5 (12.8)	6 (16.7)	0.36
BARC 3B bleeding, n (%)	3 (7.7)	4 (9.8)	4 (10.3)	5 (13.9)	0.40
BARC 3C bleeding, n (%)	1 (2.6)	0 (0.0)	1 (2.6)	1 (2.8)	0.75
BARC 4 bleeding, n (%)	0 (0.0)	0 (0.0)	0 (0.0)	0 (0.0)	-
BARC 5 bleeding, n (%)	0 (0.0)	0 (0.0)	0 (0.0)	0 (0.0)	-
Procedure-related adverse events, n (%)	0 (0.0)	0 (0.0)	0 (0.0)	0 (0.0)	-
**All-cause mortality**					
30-day all-cause mortality, n (%)	2 (5.1)	2 (4.9)	3 (7.7)	5 (13.9)	0.14

BARC, Bleeding Academic Research Consortium; USCDT, ultrasound-facilitated catheter-directed thrombolysis.

## Data Availability

The original contributions presented in this study are included in the article, and further inquiries can be directed to the corresponding authors.

## Abbreviations

BARC	Bleeding Academic Research Consortium
CTPA	Computed tomography pulmonary angiogram
eGFR	Estimated glomerular filtration rate
PA	Pulmonary artery
PE	Pulmonary embolism
PESI	Pulmonary embolism severity index
ROS	Reactive oxygen species
RV/LV	Right ventricle to left ventricle
USCDT	Ultrasound-facilitated catheter-directed thrombolysis
